# Unraveling the Functional Role of NPF6 Transporters

**DOI:** 10.3389/fpls.2018.00973

**Published:** 2018-07-10

**Authors:** Zhengyu Wen, Brent N. Kaiser

**Affiliations:** School of Life and Environmental Sciences, Sydney Institute of Agriculture, The University of Sydney, Brownlow Hill, NSW, Australia

**Keywords:** NPF6.3, NRT1.1, dual-affinity, nitrate transport, Arabidopsis

## Abstract

The nitrate transporter 1/peptide transporter (NPF) family represents a growing list of putative nitrate permeable transport proteins expressed within multiple cell types and tissues across a diverse range of plant species. Their designation as nitrate permeable and/or selective transporters is slowly being defined as more genes are characterized and their functional activities tested both *in planta* and *in vitro*. The most notable of the NPF family has been the *Arabidopsis thaliana* homolog, *AtNPF6.3*, previously known as *AtNRT1.1* or *CHL1*. AtNPF6.3 has traditionally been characterized as a dual-affinity nitrate transporter contributing to root nitrate uptake in *Arabidopsis*. It has also been identified as a nitrate sensor which regulates the expression of high-affinity nitrate transport proteins NRT2s and lateral root development as a part of the primary nitrate response in plants. The sensor function of AtNPF6.3 has also been attributed to its auxin transport activity. Other homologs of AtNPF6.3 are now being described highlighting the variability in their functional capabilities (alternative substrates and kinetics) linking to structural aspects of the proteins. This review focusses on NPF6.3-like transport proteins and the knowledge that has been gained since their initial discovery over two decades ago. The review will investigate from a structural point of view how NPF6.3-like proteins may transport nitrate as well as other ions and what can be learned from structural uniqueness about predicted activities in plants.

## Introduction

**FIGURE 1:**
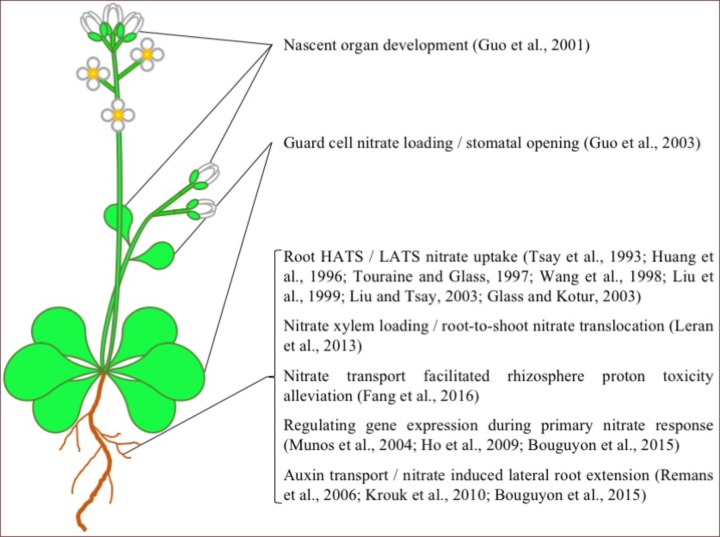
AtNPF6.3 Function in *Arabidopsis*. A list of the physiological functions of AtNPF6.3 in Arabidopsis. Picture source: http://laoblogger.com/arabidopsis-clipart.html.

**FIGURE 2:**
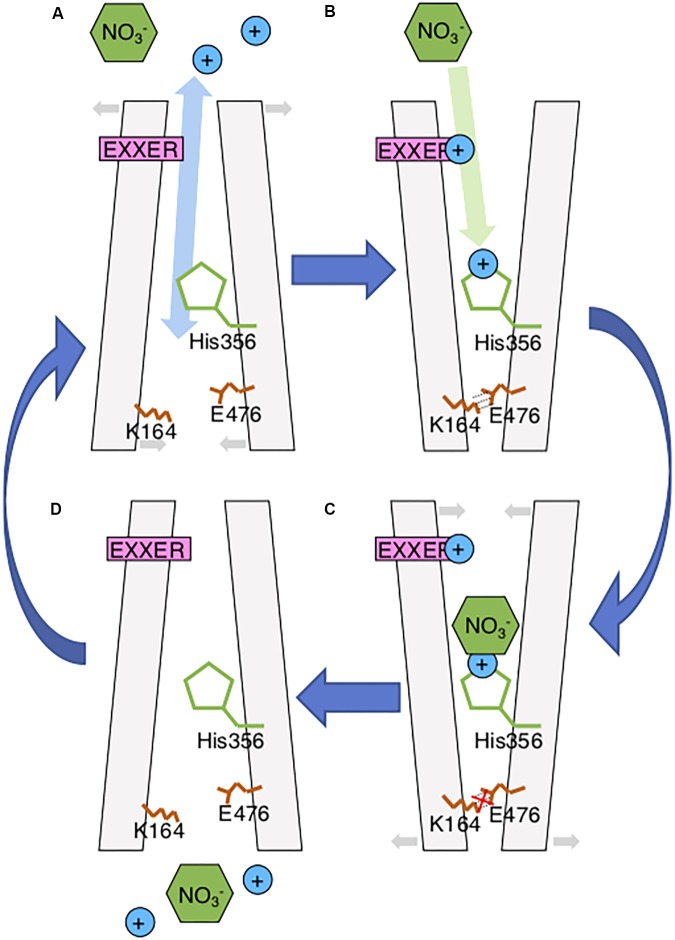
Updated AtNPF6.3 Model. **(A)** During the inward-facing stage, extracellular protons may enter and bind to the EXXER motif and His356 through a possible water molecule network observed in bacterial NPFs initiating the conformational rearrangement toward outward-facing stage; **(B)** While the salt bridge consists of K164 and E476 holding the outward-facing conformation, nitrate molecules enter the protein and bind to the protonated His356; **(C)** Nitrate-binding the His356 and disrupting the salt bridge. This causes a rearrangement of the protein toward the inward-facing stage; **(D)** Nitrate and protons are released through the intracellular gate of the protein.

Arabidopsis NPF6.3, also known as CHL1 and NRT1.1, is undoubtedly the most well studied plant NRT in the NPF family. Since NPF6.3s discovery in 1993 (Tsay et al., 1993), numerous studies have examined its *in planta* function and transport mechanism (**Figures 1, 2**). From these studies, NPF6.3 is a versatile transport protein in its substrate specificity, proposed physiological function and its location across cell types and organs. In roots, NPF6.3 has been suggested to participate directly in both high-affinity and low-affinity nitrate transport (Huang et al., 1996; Wang et al., 1998; Liu et al., 1999). Its activity across concentration gradients has been linked to the regulation of nitrate transport and assimilation genes as part of the primary nitrate response in plant roots resulting in root growth toward nitrate-rich soils (Remans et al., 2006; Ho et al., 2009). In leaves, NPF6.3 is expressed in guard cells and linked to stomata function and water use efficiency (Guo et al., 2003). NPF6.3 also transports auxin, a process negatively regulated by nitrate. The interaction between auxin and nitrate is linked to nitrate sensing and regulation of nitrate-dependent root development (Remans et al., 2006; Krouk et al., 2010; Bouguyon et al., 2015). NPF6.3 activity has also been detected in nascent organs and considered important for the development and growth of roots, stems, leaves and flower buds (Guo et al., 2001).

The structure of NPF6.3 was recently described (Parker and Newstead, 2014; Sun et al., 2014). In both models, it was concluded that NPF6.3 transports nitrate using an “alternating access” mechanism similar to bacterial homologs, where substrate transport is facilitated by conformational rearrangement between outward-facing, occluded and inward-facing stages (**Figure 2**) (Solcan et al., 2012). The transport cycle begins with the protonation of the substrate binding residue, H356, followed by nitrate binding to the protonated His356 through a charge–charge interaction when NPF6.3 is in the outward-facing stage. However, a recent study of a bacterial NPF/POT/PTR homolog (PepT_Xc_) suggested that proton and substrate binding of NPF members could involve two independent events occurring during the inward-facing and outward-facing stages, respectively (Parker et al., 2017). In its inward-facing structure, a water molecule network is observed in the central cavity extending from the substrate binding site to the ExxER motif. This could facilitate extracellular water-bonded protons entering and binding to the proton binding sites to initiate conformation change even when the extracellular gate is closed (**Figure 2**). The conformational change of NPF6.3 during nitrate transport was confirmed using FRET analysis of a fluorescent NPF6.3 sensor positioned between an acceptor and a donor fluorophore in the presence or absence of nitrate (Ho and Frommer, 2014). This FRET signal change was nitrate-specific and cannot be affected by other ions or dipeptides.

The two independent structures explain the nitrate transport mechanism of NPF6.3. They also confirm the 2:1 stoichiometry of proton/nitrate symport observed in Arabidopsis roots from physiological and electrophysiological studies (Meharg and Blatt, 1995; Miller and Smith, 1996). However, there are still questions around NPF6.3, such as its dual-affinity transport activity, auxin transport and its link to nitrate signaling. In this review, these questions are discussed with emphasis on the recent developments in NPF6.3 and its homologs.

## His356 is the Key Structural Element for Nitrate Transport

AtNPF6.3 shares conserved structural elements (an ExxER motif and an intercellular gate salt bridge) with bacterial NPF homologs. However, the His356 substrate binding residue is unique to a select number of plant NPFs (Parker and Newstead, 2014; Sun et al., 2014; Hu et al., 2015; Wen et al., 2017). His356 replaces and consolidates the function of the peptide binding/specificity pocket and the proton-binding residues located on TM7 of bacterial NPFs (Newstead, 2017). The nitrate binding activity of His356 was demonstrated by mutagenesis studies where H356A abolished nitrate transport activity of AtNPF6.3 (Parker and Newstead, 2014; Sun et al., 2014). The nitrate selective function has recently been observed in an AtNPF6.3 maize homolog, ZmNPF6.4, where replacing Tyr370 to His (equivalent to AtNPF6.3:His356) altered substrate selectivity from chloride to nitrate (Wen et al., 2017). Apart from AtNPF6.3, only two other published NPFs contain an equivalent His residue, ZmNPF6.6 and OsNPF6.5 (Hu et al., 2015; Wen et al., 2017). Interestingly, while sharing a nitrate selectivity residue, both NPFs facilitate nitrate transport at low concentrations (0.25 mM), suggesting the His residue is also important for high-affinity nitrate transport.

## Chloride Transport in NPF6s

Our group recently characterized the chloride transport activity in AtNPF6.3, and its His-containing homolog, ZmNPF6.6 (Wen et al., 2017). AtNPF6.3 and ZmNPF6.6 transport chloride when nitrate is absent, following a linear un-saturable pattern. According to Ho and Frommer (2014), chloride does not change the FRET signal of the AtNPF6.3 fluorescent sensor. Therefore AtNPF6.3 must maintain its conformation without going through an “alternative access” cycle when transporting chloride. Additionally, chloride transport in AtNPF6.3 is unlikely to involve a substrate-transporter binding episode as no chloride molecule was trapped in the AtNPF6.3 structure during crystallization even though chloride was present (Sun et al., 2014). Given the strong inhibition of nitrate on chloride transport in both AtNPF6.3 and ZmNPF6.6, we suggest nitrate occupation of the substrate binding pocket must block chloride transport (Wen et al., 2017). The chloride transport properties of AtNPF6.3 and ZmNPF6.6 indicate a channel-like activity similar to activities observed in protein families, including LacY, AKT, KUP, and CLC (Conde et al., 2010). Nevertheless, both proteins must overcome an inherent internal chloride concentration gradient (∼24–62 mM) to permit chloride uptake into *Xenopus* oocytes (Weber, 1999; Bröer, 2010). This suggests that some level of facilitated chloride transport may occur in AtNPF6.3 and ZmNPF6.6. The chloride permeability of these NPFs may provide plants a physiological opportunity for chloride uptake (Wege et al., 2017). Correspondingly, nitrate selectivity could act to inhibit excessive chloride uptake to avoid chloride toxicity (Wen et al., 2017). In addition, given the similar effect of nitrate on auxin transport in AtNPF6.3, the auxin transport activity may share a similar channel-like mechanism with that for chloride (Krouk et al., 2010). Further structural evidence will be required to confirm if this is the case.

Chloride transport has been reported in another NPF member, ZmNPF6.4, although with distinct differences (Wen et al., 2017). Unlike AtNPF6.3 and ZmNPF6.6, ZmNPF6.4 transports chloride selectively over nitrate. We believe the high-affinity chloride transport must also be facilitated by a substrate-binding residue located in the central pore of ZmNPF6.4. This mechanism would function in a similar manner to the His residue in AtNPF6.3 and ZmNPF6.6 but instead of nitrate, it binds chloride. Alternatively, the selective and binding residues could be two different structural elements, similar to those reported in bacterial NPFs. A ZmNPF6.4 protein structure will help identify the chloride transport mechanism as would a fluorescent sensor (Ho and Frommer, 2014) designed against ZmNPF6.4 to validate chloride induced conformational rearrangement during transport.

## Dual-Affinity *in Vitro*?

From a biophysical perspective, all evidence supporting dual-affinity nitrate transport activity of AtNPF6.3 (and MtNPF6.8 discussed below) are from concentration gradient nitrate flux experiments using the *Xenopus laevis* oocytes. In these experiments, nitrate concentrations were defined as high-affinity (below 0.25 mM) and low-affinity (above 0.25 mM), based on HATS and LATS nitrate uptake rates from plant studies (Glass, 2009; Wang et al., 2012). Rates of nitrate uptake were fitted using two separate Michaelis-Menten curves, resulting in two *K*m values for high- and low-affinity activities, respectively (Liu et al., 1999; Morère-Le Paven et al., 2011). We believe this method may not be appropriate for separating the two affinity ranges using a value (0.25 mM) derived from physiological studies. If the dual-affinity model is true, to observe a bi-phasic flux curve, there must be a proportion of NPF6 in the high-affinity mode and the rest in the low-affinity mode. Then, at any given concentration, nitrate uptake should be the sum of the nitrate transported through transporters in the high- and the low-affinity modes, described using a double Michaelis-Menten equation:

$$Y\;=\;a\%\times \frac{{\mathrm{VmaxHA}}^{*}X}{\mathrm{KmHA+X}}+(1-a\%)\times \frac{{\mathrm{VmaxLA}}^{*}X}{\mathrm{KmLA+X}}$$

**FIGURE 3:**
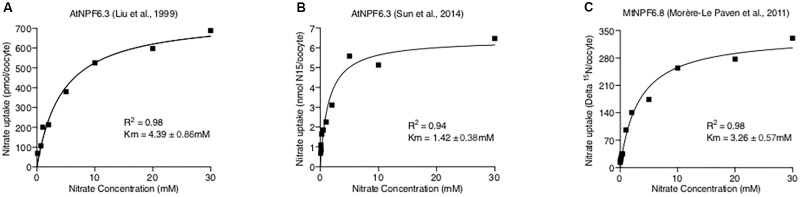
Curve Fitting NPF6.3 Comparisons. Published NPF6 dual-affinity nitrate uptake data (**A**. Liu et al., 1999; **B**. Sun et al., 2014; **C**. Morère-Le Paven et al., 2011) were directly measured from original figures and fitted with both Michaelis-Menten and double Michaelis-Menten equations using Prism 7 software. The detailed curve fitting results are listed in the tables below figures of each data set with the preferred model.

Where “*Y*” is the total nitrate uptake amount, “*X*” is the nitrate concentration, “a%” is the percentage of the high-affinity mode transporter, *V*maxHA, *V*maxLA, *K*mHA, and *K*mLA are the Michaelis-Menten constants for two action modes. However, when tested against published data of AtNPF6.3 and MtNPF6.8 (Liu et al., 1999; Morère-Le Paven et al., 2011; Sun et al., 2014), the curve fits were found to be poor (**Figure 3**). Instead, applying a normal Michaelis-Menten equation provided the best fit to the data, suggesting AtNPF6.3 and MtNPF6.8 are most-likely single-phasic nitrate transporters when expressed in oocytes. Further, kinetic studies of AtNPF6.3 by Martin et al. (2008) in yeast and our group (Wen et al., 2017) in *Xenopus* also support the single-phasic model.

## Dual-Affinity *in Planta*?

The dual-affinity nitrate transport activity of NPF6.3 as well as its net contribution to *in planta* nitrate uptake remains a controversial topic. As the activity of NPF6.3 was recently reviewed by Glass and Kotur (2013), we will only comment on a few key points about its potential activity and role. As discussed previously, experimental evidence of a dual-affinity (HATS and LATS) nitrate transport activity has been documented when NPF6.3 is expressed in *Xenopus laevis* oocytes and indirectly using the NPF6.3 knockout mutant, *chl1-5* (Liu et al., 1999; Liu and Tsay, 2003). In Arabidopsis, the evidence of dual-affinity activity linked to NPF6.3 remains limited as most nitrate transport measurements from low to high concentrations fail to see the distinct saturation kinetics required to support a biphasic transport system (Huang et al., 1996; Touraine and Glass, 1997; Glass and Kotur, 2013). Furthermore, evidence of a HATS activity and a corresponding nitrate uptake was missing in both the *nrt3.1* mutant (Okamoto et al., 2006) and the *nrt2.1* ×*nrt2.2* double mutant (Li et al., 2007), which in theory both should still express NPF6.3, and if active would display a HATS activity. Liu and Tsay (2003) do show a loss of potential HATS activity in *chl1-5* relative to a wildtype control. Unfortunately, the evidence that suggests nitrate uptake across the HATS range is in fact hyperbolic in the WT control but not in the *chl1-5* mutant is compromised by the limited range of external nitrate concentrations tested, the extended period of which nitrate influx was measured and the prior growth exposure to NH_4_^+^ which is known to influence the LATS activity in *chl1-5* (inhibiting transport when grown on NH_4_NO_3_, but not KNO_3_ (see Touraine and Glass, 1997). This lack of a strong relationship with nitrate uptake in planta suggests NPF6.3 may not have a direct role in the process, but rather a minor or targeted contribution to the overall transport of nitrate in plants.

Given the proposed nitrate sensing function of NPF6.3, a loss of activity could alter the expression of other genes responsible for nitrate uptake and assimilation, a process which may influence the nitrate uptake pathways in the *chl1-5* mutant as external nitrogen provision varies (Ho et al., 2009; Bouguyon et al., 2015). The sensor function of NPF6.3 may also help to explain the uncertainty on why *chl1-5* can tolerate chlorate even though it still able to absorb chlorate (Touraine and Glass, 1997). In the absence of NPF6.3, a disruption in nitrate-linked signaling could influence nitrate reductase activity that may alleviate toxic concentrations of chlorite being generated through the reduction of chlorate by nitrate reductase.

The nitrate uptake contribution of NPF6s has also been evaluated in other species. In dwarf maize Gaspe, the gene activity of *ZmNPF6.4* and *ZmNPF6.6* were found to be very low relative to *ZmNRT2.1* and *ZmNRT2.2* where transcript levels were 200–300 × higher and subsequently presumed the primary nitrate uptake mechanism (Garnett et al., 2013). Although the transcriptional responses of NPF6.3-like proteins in Gaspe lines suggest a limited role to either HATS or LATS activities, their functional role still needs to be resolved. Recently, we have demonstrated that ZmNPF6.6 is a significant contributor to the nitrate response, where transcript abundance is linked to increased nitrate uptake in crown roots of the maize inbred F44 relative to the common inbred B73 (Dechorgnat et al., 2018). These data demonstrate that ZmNPF6.6 may in fact be acquired for specific purposes and able to contribute to net plant nitrate uptake.

## Thr101

If we consider AtNPF6.3 as only a single-phasic nitrate transporters, then the proposed role of Thr101 as a substrate affinity switch needs to be reconsidered. Liu and Tsay (2003) suggested that when T101 is not phosphorylated (or mimicked by T101A mutation), AtNPF6.3 exhibits a low-affinity transport activity, and vice versa. In a AtNPF6.3 maize homolog ZmNPF6.6, we failed to observe similar effects of T104 mutations (equivalent to AtNPF6.3:T101) on nitrate transport (Wen et al., 2017), where both mutations kept similar kinetics with the transporter, suggesting the nitrate binding mechanism in ZmNPF6.6 was not affected. This is consistent with Parker and Newstead (2014) who show the *K*_D_ value for nitrate binding of AtNPF6.3 is not changed by the T101 mutation. However, the phosphorylation of this highly conserved Thr residue seems to have different effects to different NPF members and/or when transporting different substrates. For example, the high-affinity chloride transporter ZmNPF6.4 was converted into a low-affinity transporter by equivalent Thr mutations (Wen et al., 2017). Another example is that the dephosphorylated AtNPF6.3 loses its auxin permeability (Bouguyon et al., 2015). So far, the Thr residue has only been proposed to have an effect on the structural conformation of AtNPF6.3, either by distorting the packing of N-terminal TM bundles or disrupting protein dimerization (Parker and Newstead, 2014; Sun et al., 2014). As for how it affects the transport activity of NPF proteins this will require further study.

## NPF6 Center Loop

Another unique feature of plant NPFs is the intercellular loop linking the two six-helical bundles between TM6 and TM7. The structure of this 84-amino acid center loop in AtNPF6.3 has only been partially solved since most of the loop is disordered in crystal structures. This center loop contains three conserved positively charged residues, Arg264-Arg266-Lys267. Parker and Newstead (2014) have suggested these residues could play a role in stabilizing the protein from the intercellular side. Indeed, Ho and Frommer (2014) confirmed the importance of these structural elements where nitrate transport activity is inhibited in a R264A-R266A-K267A mutant. Sun et al. (2014) suggested that the stable amphipathic alpha-helix in the N-terminal region (including Arg264-Arg266-Lys267) of the center loop could be a potential protein-docking site. In rice, differential nitrate sensing patterns exist through natural variation in the center loop region of an AtNPF6.3 rice homolog, OsNPF6.5 (Hu et al., 2015). The authors identified that variants with the OsNPF6.5:Met327 substitution rather than those with OsNPF6.5:Thr327, influenced the primary nitrate response (nitrate uptake, root-to-shoot nitrate transport) and upregulation of the nitrate reductase genes *OsNIA1* and *OsNIA2*. Collectively, the activity of the loop region suggests a post-translational regulatory site of NPF6s and possibly an insight into the nitrate sensing mechanism?

## Bi-Directional Nitrate Transport

Previous studies suggest nitrate transport activity of AtNPF6.3 may be partially bi-directional. Leran et al. (2013) reported that AtNPF6.3 facilitated nitrate efflux in *Xenopus* oocytes, an activity later confirmed by Wen et al. (2017). These observations would suggest that the “alternating access” transport cycle of AtNPF6.3-like proteins can be reversed. Unfortunately, there is no structural evidence to support this suggestion which would require further investigation. It is interesting that NPF6.3-like proteins display both influx and efflux activities when plants already express a dedicated nitrate efflux system such as AtNPF2.7 (AtNAXT1) (Segonzac et al., 2007). Apart from structural confirmation, *in planta* estimates of bidirectional transport needs to be verified.

## Tonoplast Localized NPF6

Recently a tonoplast localized NPF6, OsNPF6.3 was characterized in rice, where overexpression enhanced both yield and nitrogen use-efficiency (Wang et al., 2018). An *osnpf6.3* knock-out mutant was able to phenocopy many of the *chl1-5* features while also exhibiting a strong repression of nitrogen assimilation associated genes. These observations would suggest OsNPF6.3 may also act as a nitrate sensor? It is already known using the uptake/sensing decoupling mutant, *chl1-9*, that the sensor function of AtNPF6.3 does not require plasma membrane targeting of the protein (Bouguyon et al., 2015). Further research is required to confirm whether tonoplast localized sensors are involved in the OsNPF6.3 nitrate signal transduction pathway and why a tonoplast location is important.

## Other NPFs

His356 is an important structural element for nitrate transport of AtNPF6.3. However, most of the NPF nitrate transporters that have been characterized do not have an equivalent His residue, or other charged residues that can bind nitrate electrostatically in the equivalent position of His356. Nitrate is then probably transported using a similar channel-like mechanism to that of the chloride transport activity in AtNPF6.3. If this occurs, then it is reasonable to question what is the correct substrate that could induce the alternative access cycle of these NPFs. It is important to stress that without examination, it is premature to conclude a NPF as a nitrate transporter. For example, AtNPF4.6 (also known as AtNRT1.2) was first characterized by Huang et al. (1999) as a constitutive LATS nitrate transporter with a low-affinity *K*m of ∼5.9 mM. However, a much higher transport affinity of AtNPF4.6 toward ABA (*K*m ∼5 μM) was reported by Kanno et al. (2012) and nitrate dose not compete with ABA transport regardless of concentration. These results suggest ABA is probably the primary substrate of AtNPF4.6, not nitrate. Physiological studies also do not agree with the nitrate transport activity of AtNPF4.6 *in vivo* as (1) nitrate has the same seed germination induction effect in both *atnpf4.6* mutants and wild type plants and (2) *atnpf4.6* mutant did not exhibit the ABA-hypersensitive effect, which would expect to happen if AtNPF4.6 was a nitrate transporters (Kanno et al., 2012, 2013). Reduced stomatal closing and associated leaf/stem surface temperature decreases were observed in *atnpf4.6* knockoff mutants, but also in overexpression mutants. Accordingly, Kanno et al. (2012) suggested a physiological role of AtNPF4.6 as an ABA pool size regulator during the biosynthesis of ABA in vascular parenchyma cells. Similarly, the role of another ABA permeable nitrate transporters, MtNPF6.8, may require further validation as it may have a nitrate sensor function regulating root architecture through its ABA transport activity (Morère-Le Paven et al., 2011; Pellizzaro et al., 2014).

Unlike the nitrate binding His residue, the structural element, ExxER, is a highly conserved motif across the NPF family (Solcan et al., 2012). It has been suggested the ExxER is an essential structural component of the proton-coupling substrate import activity in NPFs (Jorgensen et al., 2015). However, some characterized NPF nitrate transporters do not contain the ExxER motif or the nitrate binding His residue. For example, AtNPF7.3 (AtNRT1.5) lacks both elements although it was originally characterized as a bidirectional nitrate transporters expressed in root pericycle cells involved in root-to-shoot nitrate translocation (Lin et al., 2008). Recently, a second *atnpf7.3* mutant (*lks2*) was identified based on a low potassium leaf chlorosis phenotype (Li et al., 2017). The transport activity of NPF7.3 was redefined as a proton-coupled H^+^/K^+^ antiporter using the NMT/MIFE technique. Therefore, NPF7.3 may have a role in potassium translocation from root to shoot, although root-to-shoot relocation of nitrate was also reduced in the *atnpf7.3* mutant. The detailed mechanism behind this nitrate phenotype, or whether it is related to the transporter activity of NPF7.3 remains unclear. Similarly, the transporter function and physiological role of AtNPF7.2 (AtNRT1.8), which also does not have the ExxER motif or a nitrate binding His residue requires further study (Li et al., 2010).

## Perspectives

With each NPF characterization, the breadth and complexity of the transport family continues to expand. This is evident in the range of transported substrates linked to individual NPF proteins as well as their cellular localization and relevance to plant growth. What is evident is the difficulty in prescribing *in planta* function from sequence homology and current technological measurements. The recent crystallization studies examining Arabidopsis NPF6.3 identified new insights on its structural state and functional activities. New crystal structures of plant NPF proteins trapped in different conformations (outward-facing, occluded, and inward-facing) will be required to define the substrate transport cycle for each protein. When combined with existing biochemical, electrophysiological and physiological approaches the improved knowledge base will help describe the variability of new and existing NPF proteins. For NPFs, the reliance on the traditional TEVC system needs to accommodate alternative technologies including chemical flux analysis, a greater use of MIFE to evaluate the cross-membrane movement of particular cations or anions in real time (Shabala et al., 2013) and a greater use of the liposome system to describe transport activities relative to protein content (Allen and Cullis, 2013). In summary, a cross-disciplinary approach is required for plant NPF transport research to provide the necessary level of detail regarding function the community expects.

## Author Contributions

All authors listed have made a substantial, direct and intellectual contribution to the work, and approved it for publication.

## Conflict of Interest Statement

The authors declare that the research was conducted in the absence of any commercial or financial relationships that could be construed as a potential conflict of interest.

## Abbreviations

ABA: abscisic acid
HATS: high-affinity transport system
LATS: low-affinity transport system
NPF: nitrate transporter 1/peptide transporter
NRT: nitrate transporter
POT: proton-dependent oligopeptide transporter
PTR: peptide transporter
TM: transmembrane domain
